# Non-Motor Symptoms in Primary Familial Brain Calcification

**DOI:** 10.3390/jcm13133873

**Published:** 2024-06-30

**Authors:** Giulia Bonato, Paola Cimino, Francesca Pistonesi, Leonardo Salviati, Cinzia Bertolin, Miryam Carecchio

**Affiliations:** 1 Parkinson and Movement Disorders Unit, Centre for Rare Neurological Diseases (ERN-RND), Department of Neuroscience, University of Padova, 35128 Padova, Italy; 2 Center for Neurodegenerative Disease Research (CESNE), University of Padova, 35128 Padova, Italy; 3 Medical Genetics Unit, Department of Women and Children’s Health, University of Padova, 35128 Padova, Italy

**Keywords:** PFBC, COMPASS-31, non-motor symptoms

## Abstract

**Background/Objectives**: Primary Familial Brain Calcification is a rare neurodegenerative disorder of adulthood characterized by calcium deposition in the basal ganglia and other brain areas; the main clinical manifestations include movement disorders, mainly parkinsonism. Non-motor symptoms are not well defined in PFBC. This work aims at defining the burden of non-motor symptoms in PFBC. **Methods**: A clinical, genetic and neuropsychological evaluation of a cohort of PFBC patients, COMPASS-31 scale administration. **Results**: A total of 50 PFBC patients were recruited; in 25, the genetic test was negative; 10 carried mutations in *SLC20A2* gene, 8 in *MYORG*, 3 in *PDGFB*, 1 in *PDGFRB*, 2 in *JAM2* (single mutations), and one test is still ongoing. The main motor manifestation was parkinsonism. Headache was reported in 26% of subjects (especially in *PDGFB* mutation carriers), anxiety or depression in 62%, psychosis or hallucinations in 10–12%, sleep disturbances in 34%; 14% of patients reported hyposmia, 32% constipation, and 34% urinary disturbances. A neuropsychological assessment revealed cognitive involvement in 56% (sparing memory functions, to some extent). The COMPASS-31 mean score was 20.6, with higher sub-scores in orthostatic intolerance and gastrointestinal problems. *MYORG* patients and subjects with cognitive decline tended to have higher scores and bladder involvement compared to other groups. **Conclusions**: The presence of non-motor symptoms is frequent in PFBC and should be systematically assessed to better meet patients’ needs.

## 1. Introduction

Primary Familial Brain Calcification, formerly known as Fahr’s disease, is a rare adult-onset neurodegenerative disorder characterized by calcium deposition in the basal ganglia, with variable involvement of other brain areas [1].

The causative genes of PFBC include *SLC20A2* [2], *PDGFB* [3], *PDGFRB* [4] and *XPR1* [5] with an autosomal dominant inheritance, and *MYORG* [6], *JAM2* [7], *CMPK2* [8] and *NAA60* [9] with an autosomal recessive inheritance, but about 50% of patients do not carry mutations in known genes, suggesting additional, still unknown genetic contributors.

Clinical features include movement disorders, cognitive decline and psychiatric manifestations in different combinations, but patients can be asymptomatic despite extensive brain calcification. A definite genotype–phenotype correlation has not been established so far based on published cases [10,11,12,13,14,15], though some clinical and radiological features seem to be more frequent in specific genes [15], such as a higher calcification degree and more severe clinical manifestations and disease course in *MYORG* mutation carriers [16,17]. A higher risk of developing clinical manifestations in subjects with multiple brain areas involved has also been reported [14,15,16,17,18,19]; however, the relationship between the anatomical site of calcium deposition and clinical manifestations is still not defined [14,20,21].

An MDS review on 516 genetically confirmed PFBC cases has shown that 2/3 of cases were clinically affected [15]; parkinsonism was the most common motor feature, frequently associated with non-motor symptoms, including cognitive decline (in up to 50% of cases), psychiatric involvement (in up to 25%) and headache (in up to 40%), with differences in prevalence based on the underlying genetic cause [10,11,12,13,15,22].

Among additional non-motor complaints, headache has been reported as relevant in 1/3 of PFBC patients in a Japanese study, especially in *PDGFB* and *SLC20A2* mutation carriers [23], having a potential influence on quality of life and therapeutic implications [23,24,25].

The available studies have not systematically focused on non-motor features in PFBC, even in patients with PFBC-related parkinsonism, despite these symptoms (such as sleep disorders, constipation, hyposmia, orthostatic intolerance or pain) being known to be relevant in Parkinson’s disease and atypical parkinsonisms and to contribute significantly to patients’ overall wellbeing and quality of life [26,27,28,29].

This is particularly relevant for cognitive and neuropsychiatric disturbances, which are a core feature of PFBC together with motor symptoms.

For this reason, the actual prevalence of non-motor features in PFBC is unknown and may be higher and under-valued in clinical practice, with part of patients’ needs not sufficiently addressed. In fact, quality of life surveys in PFBC subjects have documented the need for an adequate support system to better accept the diagnosis, disease progression and burden for patients and their families [24,25].

This work aims at describing non-motor symptoms, including dysautonomic ones, in a single-center PFBC cohort.

## 2. Methods

Fifty patients diagnosed with PFBC and followed at the Department of Neurology of Padua University, Italy, from 2022 to 2023 were included in this study. All of them had bilateral basal ganglia calcifications on CT scan, with a Total Calcification Score (TCS) above the normal threshold for their age group [14]. Medical history and blood tests (including calcium-phosphate metabolism, muscular enzymes, and liver and kidney function) ruled out secondary causes of calcium deposition (the exclusion criteria for PFBC diagnosis, and thus for the present study). PFBC diagnosis was the sole inclusion criteria, independently from clinical presentation.

All patients signed an informed consent as needed for personal data collection and study procedures, in particular to undergo genetic testing and data sharing. No specific IRB approval was required beyond genetics consent. The study was conducted in accordance with the Declaration of Helsinki and Ethics Guidelines from the Home Institution.

Genetic testing was performed in all subjects on the DNA extracted from peripheral blood cells by Next Generation Sequencing Illumina NextSeq platform and Agilent SureSelect kit; the custom-made panel included more than 100 genes related to movement disorders and brain calcium deposition-related genes (list available upon request); ACMG (American College of Medical Genetics and Genomics) guidelines were followed for variant interpretation [30].

All patients underwent a neurological examination and an interview to assess the presence of non-motor symptoms in a qualitative fashion.

Patients were considered asymptomatic if they did not complain subjectively of any motor or cognitive symptom and if, on examination, no motor signs (such as tremor, gait disturbances, bradykinesia) or cognitive deficits were detectable. Motor symptoms were evaluated during a neurological examination by a trained neurologist (movement disorder specialist); the presence of specific neurologic and motor features (ataxia, gait disturbances, speech involvement/dysarthria, parkinsonism) was identified on clinical grounds according to the symptoms’ definition (without quantitative tools to measure motor symptoms’ severity) and the exclusion of other identifiable causes (such as orthopedic problems). Disease history and specific complaints (both motor and non-motor, as reported in Table 1 and Table 2) were collected through an interview, and were considered significant if reported by patients as constant in time and ascribable to the underlying neurological condition without other occasional explanations provided; headache was investigated when collecting patients’ clinical history by asking its features and frequency, and it was considered present and relevant based on its severity, as well as the need to take specific medication and if attacks had at least a frequency of 2/months. A neuropsychological evaluation was performed by a trained neuropsychologist, blinded to genetic results, and global cognitive functions were tested by using the Mini-Mental State Examination (MMSE) and Montreal Cognitive Assessment (MoCA) scales, adjusting the scores obtained for age and education; five cognitive domains were considered in these tests (memory, language, executive functions, visuospatial functions, attention), evaluating the presence of difficulty or errors in performing the specific tasks; a diagnosis of dementia or mild cognitive impairment (MCI) followed the current criteria, and diagnostic cut-offs were applied as per the current literature [31,32]. Behavioral features’ evaluated anxiety, depression, apathy, impulsivity (tests included Beck Depression Inventory BDI-II, Starkstein’s apathy scale SAS, State-Trait Anxiety Inventory STAI-Y 1-2, Barratt Impulsiveness Scale BIS-11, Questionnaire for Impulsive-Compulsive Disorders Rating Scale QUIP-RS, Symptom Checklist SCL-90), functional autonomy (Activities of Daily Living—ADL—and Instrumental Activities of Daily Living—IADL—scales) and quality of life (EuroQol 5-Dimension 5-level questionnaire EQ-5D-5L).

To investigate autonomic disturbances, the Composite Autonomic Symptom Scale 31 (COMPASS-31) [33,34] was administered. The questionnaire was previously validated in several neurologic disorders (Parkinson’s disease, atypical parkinsonisms, multiple sclerosis, peripheral neuropathies) [33,34,35,36,37,38]. The COMPASS-31 results were compared to a group of 18 matched idiopathic PD patients.

Statistical analysis was conducted with Chi-squared or Fisher tests for qualitative data, Mann–Whitney or Kruskal–Wallis tests for quantitative data, and Pearson or Spearman correlation tests (Jamovi).

## 3. Results

Fifty PFBC patients, 21 males and 29 females, with a mean age at evaluation of 60.9 years (±s.d. 14.6 years) and a mean age at clinical onset of 52.9 years, were included. Genetic testing resulted negative for mutations in known PFBC genes in 25 subjects (50%), whereas 14 patients carried pathogenic mutations in genes with autosomal dominant inheritance (10 *SLC20A2*, 3 *PDGFB*, 1 *PDGFRB*), 8 had biallelic *MYORG* mutations, and 2 subjects had heterozygous *JAM2* variants [39]; one had a single *MYORG* variant.

Core neurological features of PFBC (movement disorders and/or cognitive decline) were present in 76% of the cohort; overall, patients with mutations in recessive genes showed higher rates of clinical manifestations as compared to dominant gene mutation carriers (100% vs. 57%, *p* = 0.048).

Motor symptoms were found in 68% of patients; parkinsonism was the most frequent clinical finding (56%); see Table 1 for details. Dysarthria was a relevant clinical finding in 28% of the cohort, reaching a 75% frequency in *MYORG* mutation carriers (*p* = 0.04), who also had higher rates of ataxia (62.5%) compared to other sub-groups. A history of multiple falls was reported in 32% of cases, with higher rates in patients with motor involvement, especially parkinsonism or cerebellar symptoms (47%), and in those with cognitive problems (57%), or a combination of both (65%), compared to other sub-groups.

As for non-motor features, a comparison between genetic groups and clinical status was performed and symptom prevalence was documented; see Table 2 for details.

Cognitive decline was detected in 52% of the patients: 84.6% had MCI and 15.4% had dementia; the mean MMSE, adjusted for age and education, was 25.6 ± 5; the mean MoCA score was 21.6 ± 5.5. Patients mostly had difficulty in performing visuospatial and executive tasks, followed by deficits in attentive tests, whereas memory was usually less affected. MCI was more frequent in *MYORG* mutation carriers (62.5%) compared to other genetic sub-groups, with prominent difficulty in executive tasks and language functions. An association between cognitive decline and parkinsonism was observed; in fact, cognitive decline was detectable in 82.1% of subjects with parkinsonism and in only 13.6% of those without motor symptoms (*p* < 0.001), suggesting a common underlying pattern of neurodegeneration. Moreover, patients with parkinsonism had a lower mean MMSE and MoCA scores compared to patients without motor symptoms (mean MMSE of 24.1 vs. 27.9, *p* = 0.008; mean MoCA of 19.2 vs. 25, *p* = 0.002). Patients with evidence of cognitive decline of any degree had a mean TCS on the CT scan higher than patients without cognitive decline (25.5 vs. 17.3, *p* = 0.045)

Headache was overall reported in 26% of patients, but only three subjects had a migraine requiring prophylactic therapy, whereas tension-type headache was the most frequent finding. Headache was more frequent in patients carrying mutations in dominant genes and *PDGFB* subjects (66%) compared to other genetic sub-groups; no significant differences in headache prevalence could be found based on motor or cognitive symptoms.

Anxiety, depression or apathy were reported in 62% of the cohort, and psychosis or obsessive-compulsive disorder (OCD) in 10%, with a need for medical treatment in 25% of subjects. Psychiatric symptoms tended to be more frequent in patients with a genetic diagnosis compared to those without mutations in known genes (75% vs. 44%, *p* = 0.04), especially in *MYORG* mutation carriers (87.5%, *p* = 0.04). Hallucinations were reported in 12% of subjects, being mainly visual misperceptions, but also auditory hallucinations in one case. No statistically significant differences could be detected between patients with and without neurological symptoms in the overall rates of psychiatric disturbances; however, anxiety was more common in younger patients and neurologically asymptomatic subjects, shifting to depression and apathy in older patients and in those with parkinsonism or cognitive decline.

Sleep disturbances were reported by 34% of patients, with 12 subjects suffering from insomnia and 5 reporting disturbances consistent with REM Behavior Disorder (RBD). Sleep disturbances were more frequent in patients with cognitive decline compared to those without (42.8% vs. 22%, *p* = 0.04). No differences in RBD could be found in relation to parkinsonism.

Hyposmia was subjectively reported by 7 patients (14%), in association with parkinsonism in 6; there were no differences based on cognitive status or genetic etiology. Specific olfactory tests (such as UPSIT) were not administered to patients; hence, this percentage may not be fully accurate.

Constipation was reported in 32% of the cohort, with higher frequency in patients with motor symptoms (44% vs. 6.25%, *p* < 0.001), especially in those with parkinsonism or cognitive decline (*p* = 0.01) and in *MYORG* mutation carriers compared to other sub-groups, but no statistically significant differences could be detected in association with other specific symptoms or genetic etiology.

Urinary incontinence was reported in 17 patients (34%), and an additional 4 subjects reported urinary urgency, increased urinary stimulus or nycturia without daytime incontinence. Urinary symptoms were more frequent in *MYORG* patients (62.5%) and in those with either parkinsonism or cognitive decline compared to other control sub-groups (57% vs. 4.5%, 60.7% vs. 4.5%, *p* < 0.001).

COMPASS-31 was used to assess dysautonomia (see Table 3 for details on questionnaire results). The mean total score in the cohort was 20.6 (±s.d.15.4), with higher sub-scores obtained in domains exploring orthostatic intolerance (a mean weighted score of 8/40) and gastrointestinal disturbances (a mean weighted score of 5.6/25); the lowest sub-scores were detected in vasomotor symptoms (a mean weighted score of 0.5/5). Significantly higher bladder sub-scores were found in *MYORG* patients (compared to *SLC20A2* subjects and patients without known genetic variants, *p* = 0.009), as well as in subjects with parkinsonism and cognitive decline compared to asymptomatic subjects (*p* = 0.042). No significant differences could be detected in total mean score based on genetic results or among symptomatic and asymptomatic patients.

We calculated the proportion of subjects obtaining a score above 0 for each domain to identify subtle autonomic involvement (Table 3): gastrointestinal involvement was the most frequent finding (80% of patients, with a mean score of 7.07/25), followed by genitourinary problems (64%, a mean score of 3.05/10), whereas orthostatic intolerance was reported in 44% of subjects, but with higher scores (a mean score of 18.18/40). The vasomotor domain was rarely involved (16% of subjects), but it warranted a high sub-score in those affected (3.2/5). Patients with mutations in recessive genes had higher rates of positive scores in the bladder domain compared to other genetic sub-groups (100% *MYORG* vs. 64% dominant genes vs. 52% negative subjects, *p* = 0.03), as well as patients with cognitive or motor symptoms compared to those without (75% vs. 45%, *p* = 0.02). Overall, 90% of patients obtained total score above 0, and 64% had a score above 13.5/100 points, which corresponds to the threshold used to define the presence of dysautonomia in parkinsonism [34], with no significant differences among groups.

No relation between age, gender, and total COMPASS-31 scores could be found, nor between TCS, cognitive or motor scales and total COMPASS-31 scores.

Similarly, no significant differences could be found among PFBC and PD control group in both mean scores and frequency of positive answers in all the domains included in the questionnaire (Table 3).

## 4. Discussion

PFBC is a rare genetic condition characterized by calcium deposition in the brain, resulting in motor, cognitive or psychiatric manifestations.

Our study highlights that non-motor features in PFBC are present in a significant proportion of patients and may be under-diagnosed if not systematically assessed with dedicated scales and questionnaires.

Moreover, our data confirm the genetic and clinical heterogeneity of PFBC; 50% of subjects had mutations in PFBC-related genes, whereas 50% received a diagnosis of genetically undetermined PFBC. In agreement with the literature, patients with mutations in recessive genes displayed a higher disease penetrance [11,15]; motor symptoms were the most common clinical manifestation, especially parkinsonism, but with higher rates than previously reported [15]. This could be due to different data sources and acquisition methods (single center experience vs. review), or due to a selection bias, since patients were recruited from a Movement Disorders Unit. Moreover, our cohort is significantly smaller in size than previously reported cohorts, and this can justify rate variability, as well as different genetic and geographical backgrounds, as hypothesized in previous works from different cohorts [14,18,19,20].

With regard to cognitive functions, a study conducted on Dutch patients recruited in a memory clinic [40] did not support the role of calcification presence and location on cognitive functions; however, in our cohort, cognitive decline was a relevant feature, in accordance with previous prevalence studies [10,11,12,13,14,15,16,17,18], and it was also linked to a higher TCS score [20]. Cognitive problems were reported in half of our cohort, resulting in a multidomain MCI in most cases upon formal neuropsychological assessment; our data are in line with previous works describing single center cohorts in detail [14,20,21], reaching higher detection rates compared to the MDS review [15], probably due to methods used to assess cognitive decline. Even within the limits of our sample size, *MYORG* patients confirmed their distinctive and more severe phenotype, featuring dysarthria and cognitive involvement, as previously reported [16,41]. Our work remarks that cognitive decline is rarely isolated in PFBC subjects, and frequently accompanies motor symptoms, suggesting that neurodegeneration in key brain areas, such as the basal ganglia and the cerebellum, constitutes the mutual anatomical substrate of both movement disorders and dementia, resulting in a more complex clinical picture and disease management. As of now, no specific cognitive assessment tool has been validated in PFBC patients, giving rise to incomplete data with discordant results, with some papers showing prominent memory deficits [14] and others highlighting attention and executive problems [20,42]. We found that the most affected cognitive domains on MMSE and MoCA were the visuospatial ones (executive functions and attention), whereas memory functions seemed to be less involved. *MYORG* patients also had prominent language difficulties in addition to dysarthria. However, further studies on PFBC-related cognitive alterations are needed to better define specific endo-phenotypes as well as a dedicated evaluation protocol.

Psychiatric disorders are a common finding in PFBC subjects, reported in up to 1/4 of cases [15]; we detected them in 2/3 of our cohort, including mainly mood disorders but also more severe psychosis in some cases, with a need for medical treatment in 25% of subjects. Anxiety was more common in younger patients and apathy or depression in older symptomatic subjects, but no statistically significant differences could be found, probably due to the small sample size. A behavioral assessment with specific tools (BDI-II, SAS, STAI-Y 1-2, BIS-11, QUIP-RS, SCL-90) was helpful in uncovering subtle neuropsychiatric features which allowed for a better management of patients; in fact, it has been shown that the rates of mood disorder detection are greatly influenced by the accuracy of the examination [43].

As for headache, our results are in line with the MDS review [15], with almost 1/4 of the subjects affected and with higher rates for mutations in dominant genes, especially *PDGFB*. Tension-type headache was the most frequent finding, while migraine was rare; all patients required analgesic administration during attacks, usually with a satisfying clinical response, but only patients with migraine required prophylactic treatment; patients did not report significant limitations in daily activities, contrary to a recent Japanese study and survey [23,24,25]; However, we did not apply headache specific scales to assess headache severity and impact on life.

Sleep disturbances, especially RBD, are a frequent feature of PD and parkinsonism [44], but have not been previously studied in PFBC. Sleep problems were not as prominent as in idiopathic PD, and RBD was not frequent, However, overall sleep disturbances were detected in 1/3 of the cohort, mainly in subjects with cognitive decline, often requiring medical treatment.

Two papers [14,45] reported genitourinary or gastrointestinal disturbances in a small portion of PFBC patients (up to 13% of symptomatic subjects); we detected a higher rate of these symptoms, given the systematic assessment made when collecting patients’ medical history and by using the COMPASS-31 questionnaire. Constipation, one of the most frequent and bothersome non-motor symptoms of PD even in prodromal phases [46], was found in 1/3 of the PFBC cohort, especially in patients with parkinsonism. Hyposmia was also found in some patients with parkinsonism, but overall, it was a rare finding. Urinary symptoms were also reported in more than 1/3 of the cohort, with higher rates in *MYORG* patients and in those with parkinsonism and cognitive impairment.

The administration of COMPASS-31 scales for dysautonomia documented mean scores similar to PD control patients, regardless of age, sex, symptoms, genetic background and calcification extension. The domains with higher rates of complaints were gastrointestinal and bladder domain, but multiple domains were involved for each patient overall. The COMPASS-31 results also confirmed our previous results of significant urinary involvement in *MYORG* mutations and in symptomatic subjects. Overall, 2/3 of subjects had a score above 13.5, a threshold that has been used to assess dysautonomia in parkinsonism [34]. These findings document a significant overall burden of dysautonomic disturbances and non-motor symptoms in PFBC, in both symptomatic and supposedly asymptomatic subjects, thus highlighting the need for a dedicated assessment in all patients, since all non-motor features negatively affected quality of life and patients’ functional autonomy, in addition to motor features.

We acknowledge some limitations of our work, including the overall limited number of patients, especially in the sub-group analysis; however, this is justified and expected in a single-center cohort of subjects affected by a rare genetic disease; also, some possible confounders, such as patients’ age (that might influence the presence or severity of non-motor features), must be acknowledged in the interpretation of results.

Future longitudinal studies on larger PFBC cohorts will be needed to confirm our results, and to assess the efficacy of the tools used in our work, not only to individuate but also to monitor the evolution of non-motor features.

A dedicated non-motor protocol for PFBC patients could be useful in clinical practice, to harmonize the assessment tools used at present and to systematically evaluate these features that, regardless of the patients’ clinical presentation, are often overlooked and under-estimated. A prompt individuation and follow-up of non-motor features is of paramount importance to offer patients the best therapeutical options, if available, and to improve their quality of life.

## Figures and Tables

**Table 1:** Demographic and clinical features of the cohort.

	Total	SLC20A2	MYORG	PDGFB-PDGFRB	Negative Genetic Test
Number of subjects	50	10	8	4	25
Males	21 (42%)	3 (30%)	5 (62.5%)	1 (25%)	10 (40%)
Females	29 (58%)	7 (70%)	3 (37.5%)	3 (75%)	15 (60%)
Age at evaluation (y, mean ± s.d)	60.9 ± 14.6	60.3 ± 14.7	59.1 ± 8.8	52.2 ± 15.6	63.2 ± 15.8
Symptomatic (motor)	34 (68%)	6 (60%)	8 (100%)	1 (25%)	18 (72%)
Symptomatic (cognitive)	28 (56%)	5 (50%)	6 (75%)	1 (25%)	15 (60%)
Age at onset (y, mean ± s.d)	52.9 ± 13.8	51.3 ± 17	52.8 ± 8.8	45.7 ± 10.9	54.8 ± 14.5
Parkinsonism	28 (56%)	4 (40%)	6 (75%)	1 (25%)	15 (60%)
Ataxia/cerebellar features	12 (24%)	2 (20%)	5 (62.5%)	1 (25%)	4 (16%)
Dysarthria/speech disturbances	14 (28%)	2 (20%)	6 (75%)	1 (25%)	6 (24%)
Falls	16 (32%)	2 (20%)	4 (50%)	1 (25%)	9 (36%)

y: years; s.d.: standard deviation.

**Table 2:** Prevalence of non-motor features in the cohort and in different sub-groups.

	Cohort (50)	SLC20A2 (10)	MYORG (8)	PDGFB-PDGFRB (4)	Negative Genetic Test (25)	Motor Symptoms (34)	Absence of Motor Symptoms (16)	Cognitive Deficits (28)	No Cognitive Deficits (22)
Cognitive deficits: -subjective -MCI -dementia	28 (56%) 2 (4%) 22 (44%) 4 (8%)	5 (50%) 0 (0%) 4 (40%) 1 (10%)	6 (75%) 0 (0%) 5 (62.5%) 1 (12.5%)	1 (25%) 1 (25%) 0 (0%) 0 (0%)	15 (60%) 1 (4%) 12 (48%) 2 (8%)	25(73.5%) 2 (5.8%) 20(58.8%) 4 (11.7%)	3 (18.7%) 0 (0%) 2 (12.5%) 0 (0%)	28 (100%) 2 (7%) 22(78.5%) 4 (14.3%)	0 (0%) 0 (0%) 0 (0%) 0 (0%)
Domain -Memory -Executive -Visuospatial -Language -Attention	13 (26%) 17 (34%) 21 (42%) 19 (38%) 18 (36%)	4 (40%) 2 (20%) 6 (60%) 2 (20%) 2 (20%)	3 (37.5%) 6 (75%) 5 (62.5%) 5 (62.5%) 4 (50%)	0 (0%) 0 (0%) 0 (0%) 1 (25%) 1 (25%)	6 (24%) 8 (32%) 9 (36%) 10 (40%) 8 (32%)	12(35.3%) 17 (50%) 18 (53%) 17 (50%) 16 (47%)	2 (12.5%) 0 (0%) 3 (18.7%) 2 (12.5%) 0 (0%)	12(42.8%) 15(53.5%) 19(67.8%) 15(53.5%) 15(53.5%)	2 (9%) 2 (9%) 2 (9%) 4 (18%) 1 (4.5%)
MMSE (mean ± s.d.)	25.6 ± 5	25.6 ± 5	24.3 ± 4.9	27.3 ± 3.1	25.3 ± 5.8	24 ± 5.9	27.9 ± 2	23.5 ± 5.7	28.5 ± 3
MoCA (mean ± s.d.)	21.5 ± 5.5	22.1 ± 5.7	20.3 ± 5.2	22.8 ± 2.3	21.4 ± 6.4	19.1 ± 5.5	25.1 ± 2.9	18.8 ± 5.6	25.1 ± 2.7
Neuropsychiatric: Anxiety/depression Psychosis/OCD Hallucinations	31 (62%) 5 (10%) 6 (12%)	7 (70%) 0 (0%) 1 (10%)	7 (87.5%) 1 (12.5%) 1 (12.5%)	3 (75%) 0 (0%) 0 (0%)	11 (44%) 4 (16%) 4 (16%)	22(64.7%) 5 (14.7%) 6 (17.6%)	9 (56.2%) 0 (0%) 0 (0%)	18(64.3%) 5 (17.8%) 6 (21.4%)	13 (59%) 0 (0%) 0 (0%)
Headache	13 (26%)	4 (40%)	0 (0%)	2 (50%)	6 (24%)	7 (20.5%)	6 (37.5%)	5 (17.8%)	8 (36.3%)
Sleep Disturbances—RBD	17 (34%) 5 (10%)	4 (40%) 2 (20%)	3 (37.5%) 1 (12.5%)	1 (25%) 0 (0%)	7 (28%) 2 (8%)	13(38.2%) 4 (11.7%)	4 (25%) 1 (6.25%)	12(42.8%) 4 (14.3%)	5 (22.7%) 1 (4.5%)
Constipation	16 (32%)	4 (40%)	4 (50%)	1 (25%)	6 (24%)	15 (44%)	1 (6.25%)	13(46.4%)	3 (13.6%)
Genitourinary disturbances	17(34%)	4 (40%)	5 (62.5%)	1 (25%)	6 (24%)	16 (47%)	1 (6.25%)	17(60.7%)	0 (0%)
Hyposmia	7 (14%)	2 (20%)	0 (0%)	0 (0%)	4 (16%)	6 (17.6%)	1 (6.25%)	5 (17.8%)	2 (9%)
Orthostatic intolerance	3 (6%)	1 (10%)	0 (0%)	0 (0%)	1 (4%)	3 (8.8%)	0 (0%)	2 (7%)	0 (0%)
ADL (mean ± s.d.)	5.2 ± 1.5	5.2 ± 1.6	5.2 ± 1.1	5.5 ± 1	5 ±1.8	4.7 ± 1.7	5.9 ± 0.25	4.6 ± 1.7	6 ± 0
IADL (mean ± s.d.)	6 ± 2.1	7.2 ± 2.2	6 ± 1.1	6.2 ± 2	5.7 ± 2.5	5.1 ± 2.1	7.6 ± 1	5.2 ± 2	7.2 ± 1

MCI: mild cognitive impairment; MMSE: Mini-Mental State Examination; MoCA: Montreal Cognitive Assessment; OCD: obsessive compulsive disorder; RBD: Rapid eye movement sleep Behavior Disorder; ADL: Activities of Daily Living; IADL: Instrumental Activities of Daily living; s.d.: standard deviation.

**Table 3:** COMPASS-31 results.

Domain (Maximal Possible Score)	Orthostatic Intolerance (Max 40)		Vasomotor (Max 5)		Secretomotor (Max 15)		Gastrointestinal (Max 25)		Bladder (Max 10)		Pupillomotor (Max 5)		Total (Max 100)		
Group (n. of Patients)	Mean Score	% s. > 0	Mean Score	% s. > 0	Mean Score	% s. > 0	Mean Score	% s. > 0	Mean Score	% s. > 0	Mean Score	% s. > 0	Mean Score	% s. > 0	% s. > 13.5
Cohort (50)	8 ± 7.8	44%	0.5 ± 1.2	16%	3.3 ± 2.9	56%	5.7 ± 4.5	80%	2 ± 1.8	64%	1.2 ± 1.3	54%	20.6 ± 15	90%	64%
Negative (25)	8.2 ± 10.1	44%	0.7 ± 1.2	20%	3.5 ± 3	60%	6.1 ± 4.5	84%	1.5 ± 2	52%	1 ± 1.2	48%	20.9 ± 15	88%	64%
SLC20A2 (10)	6.4 ± 6	40%	0.2 ± 0.9	10%	3.2 ± 3	50%	6.2 ± 5	90%	1.4 ± 1.4	60%	0.9 ± 1.2	40%	18.4 ± 13	90%	60%
MYORG (8)	8 ± 12	37.5%	0.4 ± 1.1	12.5%	2.9 ± 3	50%	5.1 ± 5	62.5%	3.9 ± 2.3	100%	1.8 ± 1.4	87.5%	22.2 ± 18	100%	62.5%
PDGFB-PDGFRB (4)	12 ± 10	75%	0 ± 0	0%	3.2 ± 2.7	75%	4.7 ± 4	75%	2.8 ± 1.9	75%	1.9 ± 1.7	75%	24.6 ± 15	100%	100%
Parkinsonism (28)	6.4 ± 10	32.1%	0.7 ± 1	21.4%	3.1 ± 3	53.6%	5.6 ± 4.5	78.6%	2.5 ± 2.4	75%	1.1 ± 1.4	46.4%	19.5 ± 16	89.3%	57.2%
No parkinso-nism (22)	10 ± 9	59.1%	0.3 ± 0.8	9.1%	3.5 ± 3.2	59.1%	5.7 ± 4.8	81.8%	1.2 ± 1.5	50%	1.3 ± 1.2	63.6%	20 ± 14	81.8%	63.6%
Cognitive deficits (28)	7.9 ± 10.5	39.3%	0.8 ± 1.4	25%	3.5 ± 3.2	60.7%	6.5 ± 4.4	85.7%	2.5 ± 2.3	78.6%	1.2 ± 1.4	53.6%	22.4 ± 15	92.9%	64.2%
Normal cognitive tests (22)	8.2 ± 8	50%	0.2 ± 0.7	4.5%	3 ± 3.3	50%	4.6 ± 4.7	72.7%	1.2 ± 1.5	45.5%	1.1 ± 1.2	54.5%	17.1 ± 14	81.8%	59%
PD controls (18)	8.4 ± 10	42.9%	0.3 ± 0.9	14.3%	4.7 ± 4.1	76.2%	4.8 ± 3.5	85.7%	2.2 ± 1.9	76.2%	1.4 ± 1.2	71.4%	20.2 ± 18	100%	42%

s: subjects; % s. > 0: percentage of subjects scoring above 0 in the specific domain; % s. > 13.5: percentage of subjects scoring above 13.5 threshold; PD: Parkinson’s disease.

## Data Availability

The raw data supporting this study are available upon request from the corresponding author.
